# A qualitative study assessing patient perspectives in the process of decision-making on disease modifying therapies (DMT’s) in multiple sclerosis (MS)

**DOI:** 10.1371/journal.pone.0182806

**Published:** 2017-08-24

**Authors:** Archibald de Ceuninck van Capelle, Hanneke van der Meide, Frans J. H. Vosman, Leo. H. Visser

**Affiliations:** 1 Department of Ethics of Care, University of Humanistic Studies, Utrecht, the Netherlands; 2 Chair of Ethics of Care, Tilburg University, Tilburg, the Netherlands; 3 Department of Neurology, Elisabeth-Tweesteden ziekenhuis, Tilburg, the Netherlands; University of Manitoba, CANADA

## Abstract

**Background:**

Physicians commonly advise patients to begin disease modifying therapies (DMT’s) shortly after the establishment of a diagnosis of Multiple Sclerosis (MS) to prevent further relapses and disease progression. However, little is known about the meaning for patients going through the process of the diagnosis of MS and of making decisions on DMT’s in early MS.

**Objective:**

To explore the patient perspective on using DMT’s for MS. Methods: Ten participants with a recent (< 2 years) relapsing-remitting MS diagnosis were interviewed. Seven of them were using DMT’s at the time of the interview. All interviews were transcribed and analyzed using a hermeneutical-phenomenological approach.

**Results:**

The analysis revealed the following themes: (1) Constant confrontation with the disease, (2) Managing inevitable decline, (3) Hope of delaying the progression of the disease, and, (4) The importance of social support. The themes show that patients associate the recommendation to begin DMT’s (especially injectable DMT’s) with views about their bodies as well as their hopes about the future. Both considering and adhering to treatment are experienced by patients as not only matters of individual and rational deliberation, but also as activities that are lived within a web of relationships with relatives and friends.

**Conclusion:**

From the patient perspective, the use of DMT’s is not a purely rational and individual experience. More attention to the use of DMT’s as relational and lived phenomena will improve the understanding of the process of decision-making for DMT’s in MS.

## Introduction

Multiple sclerosis (MS) is a chronic and usually progressive condition that affects the brain and spinal cord [1]. It is typically diagnosed in young adults. Most patients experience the onset of their illness as the manifestation of fatigue, visual problems, bladder problems, distortion of sensory capacity, and electric sensations in limbs and spine on neck flexion. There is no definitive diagnostic test for MS. This study focuses on people with MS with a relapsing-remitting course (RRMS), which accounts for about 80% of the people who are initially diagnosed with MS [1]. Relapsing-remitting MS is characterized by periods of episodic relapses in which a sudden onset or increase in symptoms occurs, followed by a full or partial recovery. Over time, mostly around 40 years of age, 65% of the patients with RRMS enter the secondary progressive phase which may bring serious physical and cognitive disability [1]. The psychological and social consequences of MS are manifold, including increased risks of depression, divorce and unemployment [2,3]. Various qualitative studies have shown that the onset of MS is characterized by experiences of uncertainty, loss, and grief [4–6]. In addition to issues of getting used to the idea of having a chronic illness and exploring its meaning in their daily life, patients are faced with important decisions about disease-modifying therapies (DMT’s).

Although there is no cure for MS, several DMT’s have become available. Early intervention is generally recommended to limit future disease progression [7]. The most common first line DMT’s for MS in the Netherlands are interferon beta 1a (Avonex), administered weekly, interferon beta 1b (Betaferon), administered every other day, glatiramer acetate (Copaxone), administered daily or three times a week, and interferon beta 1a (Rebif), administered three times per week [8]. At the time this study was conducted, oral medications were not available; fingolimod (Gilenya)) was available in the Netherlands on open trial basis. Interferon beta 1a, interferon beta 1b, and glatiramer acetate are injectable and self-administered. The clinical efficacy of DMT’s has been demonstrated in clinical trials. DMT’s reduce the number and severity of attacks and prolong the onset of the progressive phase of MS [9]. Patients may experience side effects such as injection site reactions, flu-like symptoms, depression and arthritis. Several studies report on the relatively low percentage of patients who act in accordance with the prescribed timing, dosing and frequency of medication [10–12].

There are two strategies for treatment with DMT’s in MS: therapeutic escalation or induction therapy [9]. The idea behind therapeutic escalation is that treatment has to start with those medicines considered safest, before moving to more aggressive therapies. In induction therapy, the risk of rapidly progressive and definitive disability outweighs that of the adverse effects associated with the aggressive therapies. The decision for a specific treatment strategy is dependent on the disease activity, burden of the disease, profile of adverse events, and also the patient's preference and the neurologist's experience. The studies included in the systematic review of Michel et al. [9] show that disease activity, burden of disease, and the profile of adverse effects in relation to the use of DMT’s have been studied intensively. However, patient’ s perspectives and neurologist’s experiences as factors in decision-making on DMT’s for MS are under-researched. In this article, we address this gap by conducting an explorative study on patient perspectives on the process of decision-making on DMT’s for MS. In this paper, we set out to explore the perspectives of people with recently diagnoses MS on using DMT’s.

## Methodology

### Study design

The study followed a phenomenological research design guided by Interpretative Phenomenological Analysis (IPA). The aim of IPA is to explore in detail the processes through which participants make sense of their own experiences [13]. We believe phenomenology generally and IPA specifically can make a valuable contribution to furthering the understanding of the meaning of DMT’s for people with early MS. It draws attention to how the interpersonal, material and social contexts of the individual life shape the decision-making process on DMT’s.

### Setting and ethics

This study is part of the doctoral research project “Lived Experiences of People with Recently Diagnosed Multiple Sclerosis”. The aim of this project is to examine the lived experiences of people with MS and the meaning of their diagnosis in daily life. Earlier publications of this project described the themes of receiving the diagnosis [14], work [15] and the meaning of MS within the context of family life [16]. The ethical committee of the St. Elisabeth Hospital in Tilburg approved the research and the patients gave informed consent to participation.

### Recruitment

Inclusion criteria for this study were: (1) being diagnosed with RRMS according to the revised McDonald criteria [17]; (2) having received this diagnosis within the past two years. (3) being able to speak Dutch or English. Recruitment was carried out by a member of the interview team in spring 2012.She called patients from the records of a hospital’s ambulatory MS care to invite them to participate in our study. She contacted approximately 15 patients. Five patients declined to participate due to the following reasons: “feeling overwhelmed by the request”, “personal reasons”, and “discouraged by the psychologist”. Recruitment was stopped when the number of participants had reached ten. This number was in line with our aim of carrying out an explorative study and congruent with the demands of the IPA approach [13].

### Data collection and participants

Because we were interested in how people make sense of the diagnosis in their daily life we opted for semi-structured interviews. This method enables the participants to tell their stories and to develop their ideas and express their concerns at length. Also, it enables the researcher and participant to engage in a dialogue whereby initial questions are modified in the light of participants’ responses. We used a topic list as a way of preparing for the likely content of the interview (see S1 Table). The topic list used included the meaning of work, personal situation, diagnosis, and professional care. Following a phenomenological approach, we remained open for other topics coming up during the conversation. The use of medication emerged as an important topic in daily life as all participants discussed this extensively, whether they followed treatment or not.

Data was gathered from 13 people: ten people with MS and three partners that were asked by the person with MS to assist them with the interviews. The interviews lasted between one and two hours. All participants resided in the Netherlands and were of Western European descent. The participants with MS were aged 27–51 years. Table 1 shows the profile of the participants, ordered by age. The interviews were conducted from spring to fall 2012 by the first and the third author and a resident neurologist at the homes of the participants.

**Table 1:** Profile of the participants.

Participant	Interviewer	Gender	Age	No DMT’s	DMT’s	
					Injectable	Tablet
6	ACC	F	27		interferon beta 1a	
3	DF	F	30		glatiramer acetate	
9	ACC	F	31	V		
1	ACC	F	33	V		
5	ACC	M	35		glatiramer acetate	
10	ACC	F	41		interferon beta 1a	
8	FV	F	43			fingolimod
7	FV	F	45	V		
2	DF	M	46		glatiramer acetate	
4	DF	F	51		interferon beta 1a	

### Data analysis

Data analysis in IPA involves a ‘double hermeneutic’ because the researcher makes sense of the participant trying to make sense of what is happening to them. The analysis can be understood as an iterative and multi-directional process involving description, interpretation, processes of reduction, flexible thinking, revision, creativity, and innovation. Most IPA studies do follow a step-by-step approach in order to be systematic and rigorous, but these steps are always combined with an open attitude by the researchers. In this study, data analysis consisted of three stages of inductive analysis of the transcriptions. In the first stage, shortly after each interview, the authors and a postdoc researcher, independently from each other, added preparatory notes in Dutch in the right margins of the transcripts. Subsequently, the transcripts and notes were discussed in a plenary meeting. The cycle of interviewing, notating and discussing was repeated after each interview until the entire sample of ten interviews was completed. In the second stage, the first author systematized the notes from the first stage into a network of in vivo codes, supported by the data analysis software Atlas.ti version 6.2 (Berlin, Germany), using a constant comparison method. In the third stage, the first author used the network of codes to reconstruct a representation of patient perspectives on DMT’s in four themes. The first author made a preliminary translation of the essential quotes falling under each theme. This translation was afterwards discussed and refined with the help of a professional translator [18].

## Results

The analysis resulted in the identification of 4 interrelated themes: (1) Constant confrontation with the disease, (2) Managing inevitable decline, (3) Hope of delaying the progression of the disease, and (4) The importance of social support. Table 2 shows by which codes from which transcripts the themes are supported. This table also shows the number of related quotes that support each code.

**Table 2:** Occurrence of themes in the transcripts.

*4 themes*, *supporting codes in each transcript and number of quotes included in each code*	*Pharmacological situation*		
	*No DMT’s*	*Injectable*	*Tablet*
**(1) Constant confrontation with the disease**	P1, P9	P2, P3, P6, P10	
P1: situation 11 (6)			
P2: pain (5), medication (6), body (11)			
P3: injecting (9)			
P6: pushing the button (3), injecting (6), fear (9), medication (11			
P9: new medication (1), pricking (3), injecting (4), fear (12)			
P10: denial (3), acceptance (4), declining treatment (4), injecting (10)			
**(2) Managing inevitable decline**	P7	P4, P5, P10	
P4: abandoning injecting (1), injecting (3), medication (4)			
P5: hope (3), medication (5)			
P7: medication (4)			
P10: denial (3), acceptance (4), declining treatment (4), injecting (10)			
**(3) Hope of delaying the progression of the disease**		P4, P10	P8
P4: abandoning injecting (1), injecting (3), medication (4)			
P8: linking perceived progress with treatment (1), taking tablets (3), experimental treatment (11)			
P10: denial (3), acceptance (4), declining treatment (4), injecting (10)			
**(4) The importance of social support**		P2, P5, P6, P10	P8
P2: pain (5), medication (6), body (11)			
P5: hope (3), medication (5)			
P6: pushing the button (3), injecting (6), fear (9), medication (11)			
P8: linking perceived progress with treatment (1), taking tablets (3), experimental treatment (11)			
P10: denial (3), acceptance (4), declining treatment (4), injecting (10)			

### Constant confrontation with the disease

All participants felt caught off guard by the recommendation to begin DMT’s. This feeling was related with the mode of administration (usually injections) and the prospect of having to use medication with possible side-effects for the rest of their lives. Three participants (P3 (using an injectable), P8 (receiving oral DMT) and P9 (not using immunomodulating medication)) expressed similar feelings about using an injectable in contrast to receiving oral DMT. The idea of taking injections (initially) frightened them. The meanings behind this fear seem to vary. Some participants described how initially they struggled to find good ‘spots’ for injecting medicines and to ‘view’ their bodies from the (technical) angle of injecting medicines. P2 told in detail how he had learned with the help of his wife, to operate the injection device. P3 and P8 welcomed oral administration (through pills) as a much more ‘natural’ way to take medicines in contrast to injections. Rather than operational and technical causes of fear, they raised emotional and relational ones. The idea of taking injections (initially) made them feel different, while with oral medication it was felt possible to preserve a degree of normalcy. “*Pills*, *well*, *everybody sometimes takes a pill*, *a vitamin pill*. *But this is different*. *You have to inject yourself*. *The first time I was really shocked that I had to do this*. *But once the needle was in [my body]*, *I felt relief*. *After that*, *I started to experiment a bit*. *Now*, *injecting [medicine] is okay with me*.” {P3:295}

Besides pondering on the mode of administration, the prospect of having to use relatively invasive medication for the rest of their lives also surfaced as an element of continuous confrontation with the disease. P1 felt a ‘barrier’ to starting medication. This barrier had in her perception partially to do with not knowing how to weigh possible risks (occurrence of side effects) against possible benefits (delay of progression). The wish to become pregnant was felt by P1 as an opportunity to defer this decision as taking medication during pregnancy is discouraged. For another participant (P8), a quite ‘literal’ barrier surfaced. Having started medication, she at first no longer felt safe to travel long distances, away from her own hospital where professionals knew her and her medication. Confrontation with the disease meant for her and other participants the confrontation with a world that looked less accessible and less free. Participant 10 phrased her feeling of illness and medicines imposing limitations on her life, as follows: *“[The moment that] the doctor proposed to begin medication I thought*: *‘If I start*, *there will be no way back*. *In that case*, *I head straight towards acknowledging the fact that my life and my body are leaving me behind*.*’*” {P10:33}

### Managing inevitable decline

The recommendation of starting DMT sparked either an affirmative or negative response from the participants. Like Table 1 shows that at the time of the interview 7 of the 10 participants used medication. All participants said that they had been actively engaged with a decision about medication. Most of them perceived the limited chance to delay progression as at least *one* opportunity to do something about their situation. *“[My doctor] gave me tablets to suppress my shakiness*. *And then he proposed starting me on injections*. *I replied*: *‘If it helps*, *I consent to anything*!*’ So*, *I started injecting the medication*. *I don’t have any problem with that*, *with doing injections*.” {P4:78} Another participant, P7, perceived the limited efficacy of medication not as an opportunity for delay of progress of illness in her life, but rather as a sign of a poor product. “*Even if the efficacy had been just fifty percent*, *the choice [in favor of treatment] would have been easier*. *But it is just thirty percent*. *The efficacy of a placebo is thirty percent as well*. *That makes me think*: *‘I’ll take a Smartie instead*.*’ (…) Thirty percent*, *that is so little*.” {P7:144} Lived expectations for efficacy of medicines to manage inevitable decline hence varied through the sample. Most participants hoped to be part of the lucky 30%, for which using medicines has indeed effect.

### Hope of delaying the progression of the disease

The participants that followed the recommendation to start treatment hoped that adherence to treatment would be effective in slowing down the progression of the disease. But the sample indicated that ‘hope’ referred to more than just the statistical information about the efficacy of medicines during the entire lifespan of a patient that is part of a representative population of patients.

First, hope was also connected with perceived physical condition *here* and *now*. Participants felt motivated to continue treatment when they felt good. In the same manner, some of them felt disappointed when ‘despite’ taking medication, they went through a relapse. The following fragment illustrates how statistical and experiential knowledge about medicines can be at odds with each other: “*But then*, *the strength of my left leg began to fade*. *(…) I told him [my doctor]*: *‘I want to stop with injections”*. *He replied*: *‘Yes*, *there is only a thirty percent chance that the medication will keep you stable’*. *And yes*, *I remembered that he told me that before*. *But once my legs and my awareness began to be affected*, *I began to despair*. *‘Why do I keep going on with injections*? *It doesn’t help me anyway*.*’*” {P4:93}

Second, besides being connected to the perceived physical condition of *here* and *now*, ‘hope’ had also a relational dimension. An oral medication, fingolimod, that at the time of the interview was not yet covered by health insurances in the Netherlands, had become for participant 8 an object that signified how she worked together with her family against her disease: “*And even my family said–The medicine costs 65 euros each day*, *so that’s 25000 euros each year–last Easter*: *‘We don’t know how we will raise the money to pay for those medicines*, *but we will find a way to pay for it*, *if necessary we will pay it ourselves*.*’ I’m really lucky to be surrounded by such people*. *And that gives me hope*, *every time*.” {8:179} Clearly in this fragment it is not only the expected efficacy of the medicine that gives the participant hope. Her hope is also related to the proximity of her family. They support her in living with her disease. By paying for her medicine (or the willingness to do so if necessary), they stay with her. She’s not left on her own.

### The importance of social support

Taking or declining to take medicines was a social experience. Consideration of adhering to treatment was something that was discussed with family and friends before participants made a decision. After the decision to start treatment, family and also the MS nurse (either from the hospital or from the manufacturer of the medicine), continued to appear important in the lived experience of using medicines: “*In the beginning*, *injecting myself caused me a lot of pain*. *I have an automatic injection pen*. *It is adjustable from 0 to 10*. *But I had absolutely no clue how the button worked*. *So*, *after a month*, *I called [MS nurse] Lisa*, *because I had a couple of times injected my leg like this and the needle penetrated my leg very deeply*. *(…) Lisa explained to me how to modify the injection pen with the button and adjust it to level 4 or 6*. *So now I use level 4 for my legs and level 6 for my stomach*, *because then you can inject with more force*.” {P2:143} Other participants explained how their contact with the MS nurse from the manufacturer went beyond the technicalities of administration. They felt the call from the nurse as an opportunity to get their concerns about having MS off their chest. Participant 6 told how she had become dependent on her father and boyfriend to take her medication: “*That medication [interferon beta 1a] has to be administered by injection*. *In the beginning*, *I did this myself; the MS nurse explained to me how I had to do it*. *I was afraid and felt very nervous about it*. *Actually*, *I still do*. *I [even] don’t dare do it [injecting] myself anymore*.” {P6:121} In this and other interviews, taking medicines presents itself as a mode wherein relationships with close others (father, boyfriend, spouse) are lived.

## Discussion

Our study shows that, in the patient’s perspective, dealing with the advice to start treatment with DMT’s is a highly complicated phenomenon. Participants struggled to form personal understandings about statistical information on medical benefits and risks, and were frightened by administering injections. Once treatment had started, fear of injections and doubts about efficacy reappeared in some participants, but most of them managed to make treatment with DMT’s part of their normal daily life. The observed complexity of decision making, adjustment and adherence corroborates the findings of previous studies [19–22].

Lowden et al. view decision making on DMT’s in MS as a second step in the process of redefining the self that people with MS go through [20]. The process of redefinition starts with the disclosure of the diagnosis. It gets new momentum when people are faced with treatment options which make them reflect on their self-image (developmental, emotional, physical, and social), life and career goals, and present and future quality of life. Our themes “Constant confrontation with the disease” and “Managing inevitable decline” align with this explanation. At the outset, the injection pen presented itself to some participants as an alien device that was difficult to control. Yet, over time, participants ‘allowed’ MS and the injection pen to become part of their normal life, abandoning the pessimistic images and, guided by their own day to day experiences, developing, a more balanced perspective. This is in line with the understanding of DMT’s in MS as a matter of the redefinition of the self. Yet, the remaining two themes, “Hope of delaying the progression of the disease” and “The importance of social support” line up less well with redefining the self as the way how patients view the process of adapting to DMT’s in MS. The presence of supportive others and the presence of hope are not psychological matters. They refer to meaningful relationships and expectations about the future as two important elements in decision-making on DMT’s as a lived phenomenon. Consequently, we argue that redefinition of the self needs to be complemented by hope and the proximity of supportive others as two other essential elements in the patient perspective on decision-making on and use of DMT’s in MS.

Perhaps the most intriguing finding is the importance of social support in the use DMT’s. Five of the seven participants explained that using DMT’s was a strongly relational experience, either because of practical or financial support from family members, or because of good relations with the hospital staff and the nurses from pharmaceutical industry-sponsored patient support programs. And one of the three participants (P7) who did not use medicines at the time of the interview, also indicated that her decision *not* to take medicines had been thoroughly discussed with her family and partner. This finding corroborates other studies emphasizing the support of others as important in decision making and administration of DMT’s [19–22]. In this century, health *information* technology has become an established practice and field of research. Strongly connected with the rise of this field of research is the idea that medical decision making is (foremost) a rational enterprise and the result of interaction between the physician and the patient [23]. But our small study about DMT’s as lived phenomena within the lifeworld of patients shows a different picture. People not only think about medication, but also have feelings about it. The role of relationships that provide for the social framework wherein communication in health care flows, is largely an area that remains to be explored. Future research should investigate these relationships.

We found in qualitative research literature very little on the question of why patients discontinue DMT’s while it often occurs in practice. We only found one study that has included the perspectives of patients (n = 2) on stopping DMT’s [19]. This study shows that reasons for opting out of DMT were dislike of needles, not being “sick enough,” fear of side effects, cost versus benefit, and discouragement from physicians. In our study, just one participant (P4) had considered discontinuing because of disappointment about the efficacy of DMT. Given the estimation that the adherence rate in injectable DMT’s is sometimes as low as 49% [24], patient perspectives on discontinuation of adherence is an important issue for future research.

Several limitations to this study need to be acknowledged. First, since the only inclusion criterion was time span between diagnosis and day of the interview and type of MS (RRMS), we included participants in dissimilar pharmacological situations. This allowed us to explore diverse ways of dealing with the use of DMT’s. But it disadvantaged any deeper investigation of the meaning of specific DMT’s. Investigation of specific DMT’s as a phenomenon would be a fruitful direction for future research. Second, the results are only applicable to a specific sub-type of MS, namely RRMS. Our findings may not apply to more physically disabled people, such as patients with secondary progressive or primary progressive MS. Third, we note that the stories of the participants reflect, generally, a situation of stability in disease course and medication use. Our findings may therefore not apply to people with RRMS who experience adverse events [25], unexpected progression of disease course, treatment failure or those people who switched to other DMT’s. Fourth, being an explorative phenomenological study the sample size was relative small. In order to be able to generalize the findings, a larger qualitative study should be conducted in which saturation can be reached and in which similar themes emerge.

## Supporting information

S1 Table This is the topic list for the interviews. (DOCX)
