# Supplementary material for: A qualitative study assessing patient perspectives in the process of decision-making on disease modifying therapies (DMT’s) in multiple sclerosis (MS)
Source: PLoS One. 2017 Aug 24;12(8):e0182806. doi: 10.1371/journal.pone.0182806 (PMC5570319; doi:10.1371/journal.pone.0182806)
Supplement: S1 Table — (DOCX) [file pone.0182806.s001.docx]

# Supporting information

S1 Table.

This is the topic list for the interviews.

Theme 1: Diagnosis

You have multiple sclerosis. What happened after you heard that the diagnosis was multiple sclerosis?

Theme 2: Work or study

Did the diagnosis multiple sclerosis change your work or study?

Theme 3: Private life and leisure

Did the diagnosis multiple sclerosis change your private and social life?

Theme 4: The physician and nurse

How important is the treating physician for you? What role does the nurse play?

Can you describe the interaction between you and the physician and the nurse, specialised in multiple sclerosis?

S2 Fig. Striking image

Title: Untitled 14, Awakenings, 2015

Artwork: Hannah Laycock.

The depicted individual in this manuscript has given written informed consent (as outlined in PLOS consent form) to publish these case details. The picture (Untitled 14, *Awakenings*, 2015) was made by art photographer Hannah Laycock and is reproduced here with her written permission (License HL0017, 20/01/2017).

# Data Availability Statement

The authors confirm that, for approved reasons, some access restrictions apply to the data underlying the findings. Data excerpts are presented in the manuscript. The full dataset cannot be publicly available for ethical reasons: public availability would compromise patient privacy. Additional data may be available on request from the corresponding author, subject to seeking additional ethical approval for the release of qualitative data from the Medische Ethische ToetsingsCommissie Brabant (quote decision 23-3-2012, reference mn/12/42. Institutional contact: info@metcbrabant.nl).
